# Percutaneous subclavian artery stent-graft placement following failed ultrasound guided subclavian venous access

**DOI:** 10.1186/1471-2342-6-3

**Published:** 2006-05-05

**Authors:** Brent Burbridge, Grant Stoneham, Peter Szkup

**Affiliations:** 1 Academic Department of Medical Imaging, University of Saskatchewan, Royal University Hospital, Saskatoon, SK, Canada

## Abstract

**Background:**

Ultrasound guidance for central and peripheral venous access has been proven to improve success rates and reduce complications of venous cannulation. Appropriately trained and experienced operators add significantly to diminished patient morbidity related to venous access procedures. We discuss a patient who required an arterial stent-graft to prevent arterial hemorrhage following inadvertent cannulation of the proximal, ventral, right subclavian artery related to unsuccessful ultrasound guided access of the subclavian vein.

**Case presentation:**

During pre-operative preparation for aortic valve replacement and aorto-coronary bypass surgery an anesthetist attempted ultrasound guided venous access. The ultrasound guided attempt to access the right jugular vein failed and the ultrasound guided attempt at accessing the subclavian vein resulted in inappropriate placement of an 8.5 F sheath in the arterial system. Following angiographic imaging and specialist consultations, an arterial stent-graft was deployed in the right subclavian artery rather than perform an extensive anterior chest wall resection and dissection to extract the arterial sheath. The patient tolerated the procedure, without complication, despite occlusion of the right internal mammary artery and the right vertebral artery. There were no neurologic sequelae. There was no evidence of hemorrhage after subclavian artery sheath extraction and stent-graft implantation.

**Conclusion:**

The attempted ultrasound guided puncture of the subclavian vein resulted in placement of an 8.5 F subclavian artery catheter. Entry of the catheter into the proximal subclavian artery beneath the medial clavicle, the medial first rib and the manubrium suggests that the operator, most likely, did not directly visualize the puncture needle enter the vessel with the ultrasound. The bones of the anterior chest impede the ultrasound beam and the vessels in this area would not be visible to ultrasound imaging. Appropriate training and supervised experience in ultrasound guided venous access coupled with quality ultrasound equipment would most likely have significantly diminished the likelihood of this complication. The potential for significant patient morbidity, and possible mortality, was prevented by implantation of an arterial stent-graft.

## Background

Ultrasound has been proven to be an excellent adjunct to central and peripheral venous access procedures. [1-3] Initial publications in this area demonstrated that ultrasound guided venous access improved success rates and reduced procedure related complications. [1,2] Additionally, a recent systematic review and meta-analysis of this topic reiterated the, "clear benefit from two dimensional ultrasound guidance for central venous access compared to the landmark technique. Evidence to support this conclusion included lower technical failure rate (overall and first attempt), a reduction in complications, and faster access." [4] This review also found that the use of the Doppler capabilities of some ultrasound machines improved the success rate overall and on first attempt for the jugular vein procedures. For subclavian vein access using Doppler ultrasound, the data were in favour of the landmark technique for failed catheter placements and mean number of seconds to successful catheterization. [4]

Interestingly, this systematic review revealed quite a diversity of operator experience in the studies used to accumulate data for analysis. Operators included radiologists, anesthetists, surgical oncology fellows, junior house staff and senior intensivists, to name a few. The was no analysis of the data to determine if operator experience, or training, had any influence upon success rates and complications. [4] Common sense dictates that adequate training and experience are pivotal for the provision of safe and effective health care.

## Case presentation

This 77 year old woman was being prepared for an aortic valve replacement and tandem aorto-coronary bypass graft surgery. Under general anesthetic, the anesthetist used a Site-Rite 3 (Bard Access systems, Salt Lake City, UT, USA) ultrasound unit while attempting to access the right internal jugular vein for venous sheath placement. This attempt failed.

The anesthetist then used the same ultrasound unit attempting to access the right subclavian vein for venous sheath placement. Following what was presumed to be successful ultrasound guided venous access, an 8.5 F sheath was implanted. The anesthetist noted an arterial wave-form arising from the sheath tracing. It was presumed that the subclavian artery, beneath the mid-portion of the clavicle, had been inadvertently catheterized. The sheath was left in situ due to concerns about uncontrollable arterial bleeding if it was extracted without the ability to directly compress the arterial puncture site.

The cardiovascular surgeon performed aortic valve replacement and aorto-coronary bypass surgery. Consultation with a peripheral vascular surgeon was sought. The peripheral vascular surgeon suggested that rather than perform a complicated surgical procedure to extract the sheath, i.e. an extensive anterior chest wall dissection and resection, that interventional radiology be consulted about angiography and the possible placement of a stent-graft in the infraclavicular subclavian artery. The cardiovascular surgeon consulted the interventional radiology team.

The patient was transferred to the angiography suite directly from the operating room and remained under general anesthetic. Consent for the procedure was obtained from the patient's family.

**Figure 1:**
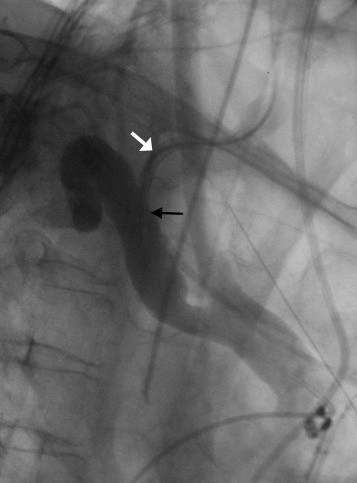
Right innominate artery angiography in right anterior oblique (RAO) 65 degree obliquity. The subclavian sheath is opacified with a small amount of contrast within the lumen of this device (white arrow). The image demonstrates the ventral, proximal entry point of the sheath into the subclavian artery (black arrow).

**Figure 2:**
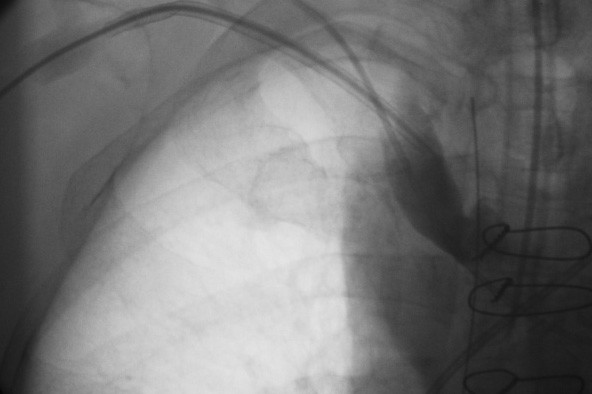
Contrast was injected via the 8.5 F sheath. An early phase image demonstrates that contrast enters the proximal subclavian artery. No extravasation noted.

**Figure 3:**
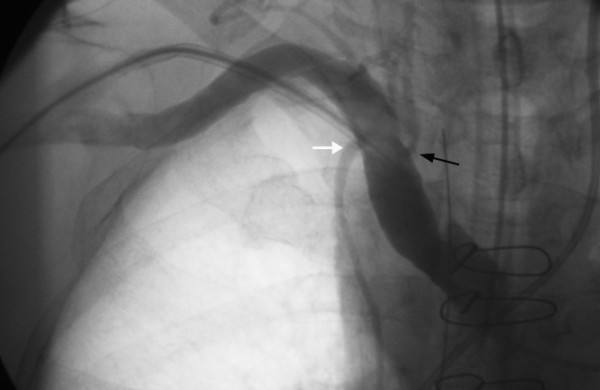
Contrast was injected via the 8.5 F sheath. A late phase image demonstrates the right internal mammary artery (white arrow) and the right vertebral artery (black arrow).

Right femoral artery access was achieved and a 6 F groin sheath was placed for angiographic imaging. Injections of the right innominate artery demonstrated a normal infra-clavicular subclavian artery. The 8.5 F sheath was situated with the tip just downstream from the origin of the right common carotid artery within the proximal right subclavian artery. (figure 1) Further angiographic images were obtained in other projections. In addition, the 8.5 F sheath was directly injected with contrast agent in order to determine the exact location of the sheath. Early (figure 2) and delayed (figure 3) angiographic images were acquired.

It was determined that the 8.5 F sheath had actually entered via the ventral aspect of the right subclavian artery just downstream of the origin of the right common carotid artery and had not punctured the more distal infra-clavicular subclavian artery as suspected by the clinical team. There was no evidence of spasm or extravasation of contrast at the site of the catheter entrance into the subclavian artery, or elsewhere. Despite the very proximal entry point of this catheter into the subclavian artery the cardiovascular surgeon had not been able to directly visualize it, or palpate it, during the surgical exploration of the mediastinum.

Right sided pelvic angiography was performed to ensure that the pelvic arterial anatomy would accommodate the placement of a stent-graft. There were no pelvic or distal aortic impediments to stent-graft placement.

The 6 F right femoral artery sheath was removed and a 12 F right common femoral artery sheath was implanted to accommodate the stent-graft. An exchange length Amplatz guide wire was situated with the tip in the right axillary artery. A 70 mm long/12 mm diameter Wallgraft endoprosthesis (Boston Scientific, Natick, MA, USA) was successfully implanted in the right subclavian artery while the 8.5 F sheath in the subclavian artery was simultaneously extracted.

**Figure 4:**
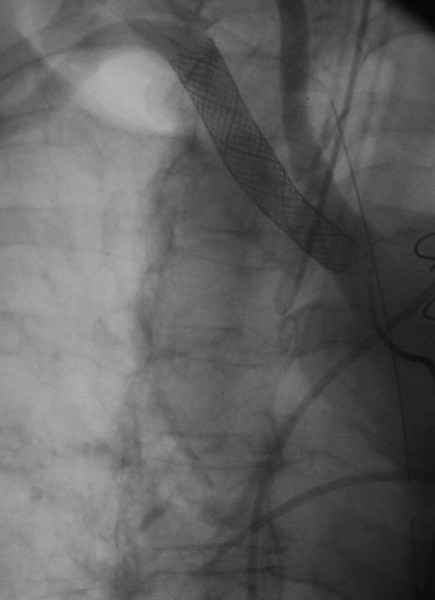
Subclavian artery angiography in RAO 25 degree obliquity. The Wallgraft has been successfully deployed. The right internal mammary artery and the right vertebral artery are occluded. The right common carotid artery is patent and normal in appearance.

Immediate post-implantation angiography demonstrated a patent stent-graft and acute occlusion of the right internal mammary artery and the right vertebral artery. There was no evidence of contrast extravasation and no clinical evidence of subclavian artery bleeding. (figure 4) The patient remained hemodynamically stable. The right groin sheath was extracted and groin hemostasis was achieved. The patient was transferred to the Intensive Care Unit (ICU) for further care. Right arm and right leg pulses were all palpable and there was no clinical evidence of hemorrhage.

The patient spent 3 days in the ICU and was then discharged to the ward. She recovered with no evidence of peripheral vascular or neurologic sequelae. She was discharged from hospital to further convalesce 3 days after leaving the ICU.

## Discussion

The ventral, and proximal, entry point of the vascular sheath into the subclavian artery raised some very interesting clinical scenarios. In order to surgically extract the catheter and control any subsequent hemorrhage an extensive resection of the manubrium, medial clavicle and medial first rib would be required. Consultation with a peripheral vascular surgeon determined that this could be performed, but the consequent morbidity and mortality due to the nature of the proposed surgery and the extension of an already protracted general anesthetic (6 hours), made this a very unattractive alternative.

Stent-graft placement was proposed. Analysis of the images determined that the right internal mammary artery and the right vertebral artery would be occluded by placement of a stent-graft in this region. There was also some concern that the origin of the right common carotid artery could be occluded if the stent-graft was not precisely placed.

Consultation with the cardiovascular surgeon confirmed that the right internal mammary artery had not been used for any of the coronary artery bypass grafts. Left subclavian angiography revealed a large left vertebral artery, larger than the right, with no abnormalities of the left vertebral artery seen.

Discussion surrounding the use of a percutaneous arterial closure device also took place. However, it was felt that the very deep entry point into the artery in relationship to the skin entry site made the use of one of these devices less attractive. In addition, if the closure device failed the clinical scenario of uncontrollable bleeding would be of concern.

It was determined that a subclavian artery stent-graft was the best treatment option given the circumstances. The coronary grafts would not be at risk and the dominant left vertebral artery made the possibility of spinal artery or posterior fossa infarction less likely.

Review of the Site-Rite 3 ultrasound system used for the patient in question revealed the lack of Doppler imaging capabilities. In addition, the machine had no capacity to store or print static images for documentation of the vascular puncture procedure. We feel that ultrasound equipment used for venous access procedures should be capable of providing Doppler images. It is the responsibility of the operator to document placement of the exploring needle within the target vein by capturing static images of the procedure. The operator should also submit a dictation or a note to the medical record accounting for the events of the venous access procedure. [5]

Ultrasound imaging is a very accurate method of safely puncturing venous and arterial structures. The goal of ultrasound guidance is to enter the skin at an appropriate location and to directly visualize the exploring needle tip enter the targeted vascular structure. The operator must be adequately trained and have sufficient experience to execute these procedures to achieve the desired increased technical success and reduced procedural complications in comparison with anatomic localization techniques.

The skin entry site for ultrasound guided venous access procedures in the infraclavicular region must be modified to suit this technique. Both Galloway, et al, and Sharma, et al, have provided detailed justification for, and validation of, a more lateral approach to the infraclavicular venous structures when ultrasound is used to guide venous puncture. [6,7] The lateral approach allows for diminished risk of arterial puncture and pneumothorax. In addition, if the artery is inadvertently punctured the operator can safely, digitally, compress the puncture site as it is more lateral and is easily compressible. This obviates the need for a surgical closure of the medially situated arterial injuries associated with the anatomic landmark technique. This lateral access site also allows for unimpeded visualization of the needle as it enters the target vein. Utilizing a medial approach, based upon anatomic landmarks, prevents visualization of the needle tip as it descends beneath the clavicle and manubrium. Adherence to these principles may have prevented the inadvertent catheter placement in this particular case.

Needle visualization is the ultimate goal of ultrasound guided venous or arterial punctures. Most likely, clinicians using ultrasound to facilitate these punctures have limited experience with ultrasound during their professional development. Chapman et al have written an excellent review article pertaining to the practical aspects of needle visualization during ultrasound guided procedures. This is a very operator dependent process and success ultimately depends upon the ability of the operator to appropriately align the ultrasound probe and the exploring needle to best advantage. The physics and physical aspects of this elegant technique are well described in this review article. [8]

The value of supervised clinical training and experience cannot be overestimated in relation to ultrasound guided interventions. These are very operator dependent procedures and considerable training in these techniques, although not scientifically validated, is without a doubt pivotal in ensuring procedure success while minimizing complications. To this point, the Royal College of Radiology (RCR) has issued a treatise outlining the training processes that should precede unsupervised clinical application of ultrasound guided interventions by non-imaging medical and surgical specialists. [9]

The RCR has created detailed guidelines for a variety of clinical specialties that use ultrasound for clinical care. These guidelines provide concise protocols for acquiring knowledge, training, experience, and defined competencies. It is expected that the trainee will work under the supervision of a qualified trainer and should document the duration and content of his/her learning experience, including a written record of exposure to the discipline and successful completion of competencies. There are no other such guidelines that we are aware of in Europe or North America. The RCR has set a standard for other professional organizations to follow.

Extraction of the incorrectly placed catheter in this situation could have led to significant complications, including this patient's demise from exsanguination or massive uncontrolled mediastinal hematoma formation. Stent-graft placement resulted in a satisfactory clinical outcome. Appropriately trained operators, applying competencies achieved through training and experience, using quality ultrasound equipment will maximize their potential for success and minimize the potential for complications related to ultrasound guided interventions in the future.

## Competing interests

The author(s) declare that they have no competing interests.

## Authors' contributions

BB – primary author, operator for stent-graft placement, approved the final manuscript.

GS, PS – read, revised and approved the final manuscript.

## Pre-publication history

The pre-publication history for this paper can be accessed here:
